# TLR2 and TLR4 Signaling Pathways and Gastric Cancer: Insights from Transcriptomics and Sample Validation

**DOI:** 10.52547/ibj.26.1.36

**Published:** 2021-11-14

**Authors:** Saeed Zargari, Abbas Bahari, Mohammad Taghi Goodarzi, Minoo Mahmoodi, Reza Valadan

**Affiliations:** 1 Department of Biology, Hamedan Branch, Islamic Azad University, Hamedan, Iran;; 2 Research Institute of Modern Biological Techniques, University of Zanjan, Zanjan, Iran;; 3 Department of Biochemistry, Shahrood Branch, Islamic Azad University, Shahrood, Iran;; 4 Department of Immunology, Molecular and Cell Biology Research Center, School of Medicine, Mazandaran University of Medical science, Mazandaran, Iran

**Keywords:** Inflammation, Gastric cancer, Gene expression, Pattern recognition receptors, RNA-seq

## Abstract

**Background:**

Pattern recognition receptors, especially TLRs, as the first line of defense for pathogen detection, were found to be associated with *H. pylori *infection and GC. However, the expression levels of TLRs, i.e. TLR2 and TLR4, as the main receptors sensed by *H. pylori*, still remain largely ambiguous. We aimed to investigate the patterns of key transcripts of TLR2 and TLR4 in 100 GC transcriptome data. Additionally, we evaluated TLR2 and TLR4 gene expressions in gastric biopsies of Iranian GC patients, in order to validate RNA-seq outputs.

**Methods:**

For this study, 100 runs of GC samples and controls were processed and analyzed using map read to reference. DGE method was used to distinguish between GC and normal samples in the expression of TLRs and other innate immune molecules. Also, using qRT-PCR assay, transcripts of TLRs molecules for 15 GC and 15 control samples were analyzed based on the analysis of variance and least significant differences.

**Results:**

The results clearly showed that all signaling pathways molecules of TLR4, especially TLR4 (*p* = 0.019), NF-κB (*p* = 0.047), IL-1β (*p* = 0.0096), and TNF-α (*p* = 0.048), were upregulated in a cancerous condition in different parts and at various stages of GC.

**Conclusion:**

Our findings suggested that molecules involved in inflammation, including TLR4 and its related pro-inflammatory cytokines, may be responsible for the development and progression of GC. Accordingly, the control of* H. pylori* infection reduces inflammation in the gastric system and can play an important role in preventing gastrointestinal disorders.

## INTRODUCTION

Gastric cancer is the fourth most common cancer and the second leading cause of cancer death worldwide^[^^1^^]^. The innate immune responses are the first line of defense against various pathogens, including bacteria, viruses, protozoa, and fungal agents. Innate immune system plays a critical role in the identification, processing, and delivery of immunogenic compounds to adaptive immunity^[^^2^^]^. 

Inflammation is a type of immune response induced under various abnormal conditions, including infections and tissue damage. Bacterial infections, as a main cause of inflammation, can be recognized by the innate immune system, and give rise to severe immune responses^[^^3^^,^^4^^]^. Although these responses are initiated by a variety of PRRs, the role of TLRs has more frequently been documented compared to others^[^^5^^]^. Pathogens identified by the innate immune system are specific, and the external constituents of these pathogens, namely PAMPs, are identified by PRRs^[^^6^^]^. Evidence has suggested that these receptors are responsible for the identification of endogenous molecules released from damaged cells called DAMPs^[^^7^^]^. Following the detection of PAMPs, many changes occur in the expression of genes, and signaling pathways would result in appropriate immune responses. Accordingly, most of these pathways could activate transcription factors such as NF-κB^[^^8^^]^.

Conserved pattern of bacterial, fungal, viral, and parasite antigens can be sensed by PRRs, especially TLRs^[^^9^^]^. Correspondingly, TLR4 activation has been found to promote (NF-κB) signaling pathway, followed by pro-inflammatory cytokine secretion^[^^10^^]^. The most well-known ligand for TLR4 is LPS originated from Gram-negative and some Gram-positive bacteria. Besides, some endogenous proteins, e.g. heat shock proteins, have an affinity to this receptor and also to co-stimulatory molecules known as LY96 or CD14, which is necessary for LPS responsiveness^[^^11^^]^. Unlike *E. coli* LPS, *H. pylori* LPS selectively upregulates IL-18 and IL-12, the main immunosuppressive cytokines especially T-cell suppression and, consequently, facilitates GC initiation and progression^[^^12^^]^. TLR2 is mainly expressed on the surface of all immune cells, and similar to TLR4, it can recognize bacterial (mostly Gram-positive) PAMP, as well as fungal, viral, and certain endogenous proteins^[^^7^^]^. Depending on the site of the infection, long-term carriage of *H. pylori* by gastrointestinal increases the risk of gastric disorders. For instance, *H. pylori* infection is responsible for ~10% of peptic ulcers and 1-3% of gastric adenocarcinoma^[^^13^^]^. However, cigarette smoking and alcohol abuse are the two other risk factors known for adenocarcinoma^[^^14^^]^. As *H. pylori *plays a key role in the development of inflammation, gastric ulcers, and GC, the role of PRRs, especially TLRs, is very important in this regard. Notably, TLR2 and TLR4, due to their bacterial ligands, are more important than other members^[^^15^^,^^16^^]^. Activation of TLR4 by *H. pylori* initiates a series of inflammatory processes in the gastrointestinal tract and also elevates TLR4 expression^[^^17^^]^. It has also been shown that TLR2 is upregulated in bone marrow dendritic cells after being exposed to *H. pylori* infection. Therefore, infection mediates immune tolerance in dendritic cells through TLR2-derived signal^[^^18^^]^. 

Owing to the controversy over the correlation between TLR2, TLR4, NF-kB, and pro-inflammatory cytokines, RNA-seq data analysis of GC is valuable for better understanding of their relationship. The main objective of the present study was to find changes in the expression pattern of TLR2 and TLR4 receptors in the GC samples extracted from the NCBI database (https://www.ncbi.nlm.nih.gov/). To confirm the results of the GC transcriptome analysis, GC biopsy specimens were collected from hospitals, and aforementioned expression pattern was then analyzed using qRT-PCR.

## MATERIALS AND METHODS

**Specimens**

 The study included 30 paraffin-embedded gastric tissues, including 15 pathologically confirmed *H. pylori*-positive GC based on urease activity in biopsy specimens (8 men and 7 women; aged 47-65; average = 60.14; SD = 4.73) and 15 control samples (9 men and 6 women; aged 50-79; average = 65.42; SD = 8.48) were used. Normal gastric biopsies from healthy individuals were selected as a control group.

**RNA-seq data analysis**

**Fig. 1:**
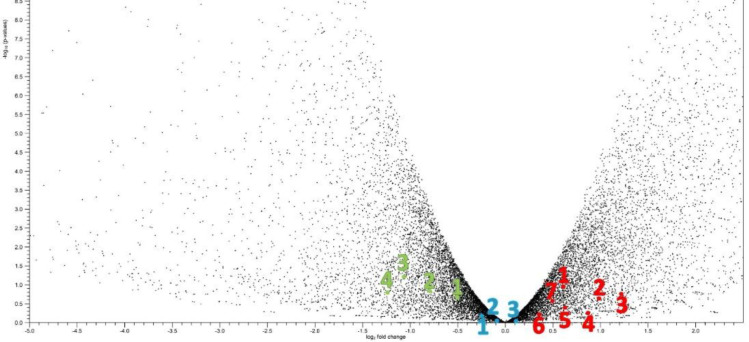
SRRs of GC and control groups collected from NCBI/SRA and mapped reads to references using CLC Genomic workbench. DGE statistical method was used to differentiate human genes in GC and control groups. Volcano graph shows the gene expression of 28,000 human transcripts in 60 GC versus 40 normal samples. Each dot in the right and the left side represents up and downregulated genes, respectively. Red, blue, and green dots represent upregulated, downregulated, and unchanged expressions, including 1-7, IL-1β, TNF-α, NF-κB, TLR4, MD2, CXCR7, and CXL12 for red ones; 1-3, IL-6, TLR2, and IL-12 for blue ones; 1-4: IL-11, IL-10, IL-13, IL-4 for green ones, as representative immune genes involved in GC

For this study, 60 runs of GC and 40 runs of normal gastric samples were downloaded in the NCBI SRA using SRA Toolkit package (Supplementary Table 1). The quality of the obtained data was analyzed by the FastQC software (https://www.bioinformatics. babraham.ac.uk/). Thereafter, those reads with low-quality scores were removed, and all adapter sequences were trimmed using CLC genomics workbench 21 (https://digitalinsights.qiagen.com/). Afterward, RNA-seq data were mapped to human ref-seq of genomes, genes, and transcripts were obtained from Ensemble (https://asia.ensembl.org/) with the following parameters: mismatch cost: 2, insertion cost: 3, and deletion cost: 3, length fraction: 0.8. Maximum number of hits for a read was set as 50. Of note, at this stage, RPKM was calculated for each SRR. The results of the mapped reads were classified into two groups, the GC versus the control, which were finally compared with each other using the DGE statistical method. For each gene, *p*value less than 0.05 and fold change more than $\left|\left|\pm 2\right|\right|$ were considered as significant differences. Scatter and volcanic curves were plotted based on the distribution pattern of the gene expression data (https://digitalinsights.qiagen.com/), as shown in Figure 1.

**RNA extraction**

For the RNA extraction, 1 ml of the extraction buffer solution (RiboEx^TM^, Gene All, South Korea) was added to 0.2 g of the samples homogenized in liquid nitrogen. The homogenate was then incubated at room temperature for 5 minutes. Subsequently, 200 μl of chloroform was added to each sample, which was then shaken for 15 s. The samples were centrifuged at 15294 rcf at 4 °C for 15 minutes. Next, the upper phase was transferred to a new microtube, and 0.5 ml of cold isopropanol was added to each sample. The solution was gently mixed and incubated at room temperature for 10 minutes. The microtubes were then centrifuged at 15294 rcf at 4 °C for 10 minutes. Following the removal of isopropanol, 1 ml of 75% ethanol was added to the samples, and after 10 s of shaking, the microtubes were centrifuged at 5974 rcf at 4 °C for 5 minutes. After removing the ethanol, the samples were left at room temperature for 30 min until the RNA pellets got relatively dried. Ultimately, 50 μl of nuclease-free water was added to each sample and then stored at -80 °C until reverse transcription steps. RNA integrity and quality were evaluated by agarose gel electrophoresis and using a NanoDrop (R&D, USA), respectively. The degraded RNA bands and samples having poor OD parameters (260/280 and 260/230) were re-extracted. GAPDH gene was used as an internal reference gene. 

Primers were designed based on the ref-seq mRNA sequences for TLR2, TLR4, NF-κB, GAPDH, TNF-α, and IL-1β using Allele ID ver.7 software (http://www.premierbiosoft.com; Table 1). In this regard, 2 µg of total RNA was applied in reverse transcription reaction in a final volume of 40 µl using RevertAid First Strand cDNA Synthesis Kit (Fermentas, Finland) and 10 pmole of oligo(dT) primer. Thereafter, all cDNA samples were diluted three times and then stored at -20 °C until qRT-PCR assay. Real-time PCR was performed in 20 µl of total reaction volume containing 500 ng of cDNA as template, 5 pmol of each of the desired primers, and 4 µl of 5X HOT FIREPol® EvaGreen® qPCR Mix Plus (Solis BioDyne, Stonia).

In the end, qRT-PCR was performed in a Rotor-Gene 6000 (Venlo, Netherlands) with a program set at 95 °C for 15 min, as the pre-denaturation step, followed by 45 cycles at 95 °C for 20 s, at 55-65 °C for 20 s (dependent on primers), and finally at 72 °C for 20 s. Melting step was programmed to confirm the reaction’s specificity. Furthermore, RT minus sample was used in qRT-PCR to check the possible genomic DNA contamination. For each biopsy sample, we performed two experimental repeats (two RNA extraction) and three technical repeats (in RT-PCR). At first, each Ct belonging to the target gene was normalized to its corresponding reference gene. Thereafter, the lowest amount of Ct values was presumed in different replications and treatments as one and the rest values were calculated relative to it. Due to this reason, all averages in bar charts show numbers greater than one. Other types of standardization of Cts, depending on the need of this research, were performed by GenEx v6 software, which included interpolate calibration, normalization to sample amount (normalization based on the initial cDNA concentration), and test for outlier (to find the Ct values considered out of data). The expression values were calculated as per the Pfaffle methods^[^^19^^]^:

$\mathrm{ratio}=\frac{{\left(E\mathrm{target}\right)}^{\mathrm{Delta}\mathrm{Ct}\mathrm{target}(\mathrm{Ctrl}-\mathrm{Sample})}}{{\left(E\mathrm{reference}\right)}^{\mathrm{Delta}\mathrm{Ct}\mathrm{ref}(\mathrm{Ctrl}-\mathrm{Sample})}}$

**Table 1:** Accession numbers, primer sequences, expected PCR fragment sizes, and annealing temperature (Ta) of the designed primer

**Target**	**Accession number**	**Forward and reverse sequences**	**Amplicon** **(bp)**	**Ta (** **°** **C)**
NF-Ƙb	NM_001007531.3	TGCTGGAGTTCAGGATAAC	179	60.7
		GGATGATTGCTAAGTGAGAC		60.7
				
TLR2	NM_001318790	GCAGGGCATGGTGGCATGTG	123	71
		CCAGGCTGGAGTGCAGTGGT		71
				
TNF-α	NM_000594.4	CTCTTCTCCTTCCTGATC	190	50.5
		CTTGAGGGTTTGCTACAA		52.00
				
IL1β	NM_000576.3	CTTTGAAGAAGAACCTATCT	178	49.7
		CACTTGTTGCTCCATATC		50.4
				
TLR4	NM_003266.4	TGGAGGACTTTAAGGGTTAC	104	55.3
		GATGTCTGGGTCTTGGTT		53.9
				
GAPDH	NM_001357943.2	GGTCAGATCCACAACGGACA	196	59.6
		CACTGCCACCCAGAAGACTG		60.6

All primers were designed based on exon-junction strategy using AllelID 7.

**Statistical analysis**

The relative expression was analyzed by SPSS ver22 software for each replication and each treatment. Moreover, the assumptions of analysis of variance, including homogeneity variance and normality test, were calculated for expression values. In many cases, logarithmic data transformation was used to achieve a normal population. The analysis of variance was performed using linear GLM models. Mean comparisons were performed by the least significant differences method. In addition, Minitab v17 software was used to group the means. A significance level of less than 5% was considered significant for means comparison.

**Ethical statement**

The above-mentioned sampling protocols were approved by the Ethics Committee of Islamic Azad University of Hamedan, Hamedan, Iran (ethical code: 4561). 

## RESULTS AND DISCUSSION

Understanding the link between microbial signals and GC can be considered as an important issue to fight against gastrointestinal cancer, improve lifestyle, and use natural anti-inflammatory substances^[^^20^^]^.

*H. pylori* can reside in a host for years, decades, or even for lifelong. This bacterium has been announced as the first cause of gastric tumorigenesis by the World Health Organization^[^^21^^]^. The positive correlation between *H. pylori* and GC has been ascribed to the components of *H. pylori*, including LPS, flagella, vacuolating toxin A and cytotoxin associated gene pathogenicity island. These agents are proposed for the pathogenicity of the *H. pylori* through changes in host gene expression, infection-induced cell proliferation, epithelial cell elongation, degradation of cell-cell junctions, and decreased gastric acid secretion^[^^22^^]^. LPS is one of the most important virulence structures of *H. pylori* in gastric environment^[^^23^^]^. Therefore, dendritic cells and other immune cells resided in the epithelial cells of the gastrointestinal system are exposed to this pathogen^[^^24^^]^. Thus, PRRs in these cells could help in permanently detecting the presence of DAMP and PAMP as well as activating the inflammatory pathways in these cells^[^^25^^]^. TLRs, especially TLR2 and TLR4, are among the most reported receptors that sense bacterial components. Notably, chronic atrophic gastritis with intestinal metaplasia was found to be associated with the increased incidence of GC compared with the normal population^[^^26^^]^.

**Fig. 2:**
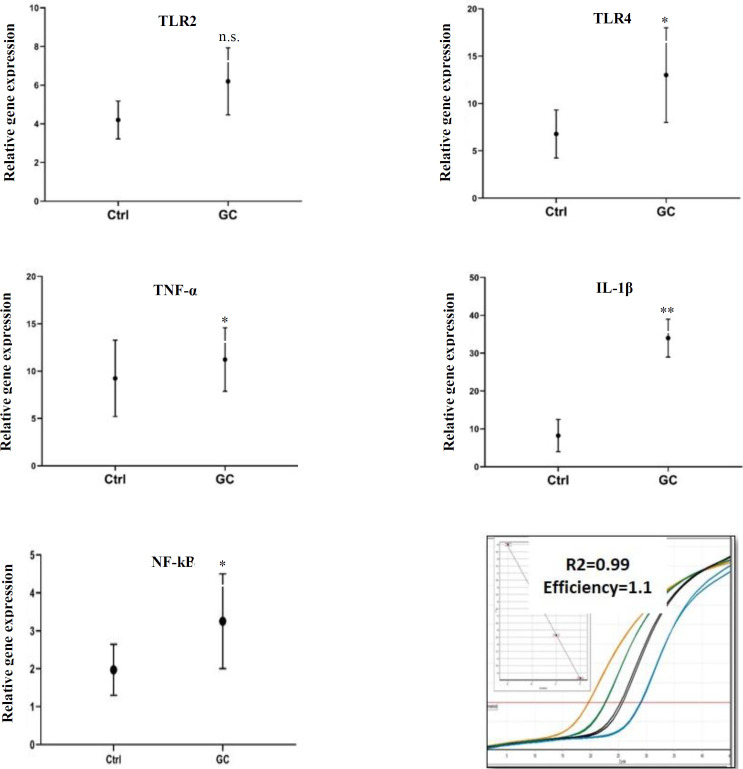
Gene expression quantification of five key molecules involved in immune innate responses to GC. All these genes statistically were overexpressed in the GC compared to the control group (except for TLR2). All the data were normalized with GAPDH as an internal control. The data are presented as the mean ± SD (n = 6). Means and error bars represent two experimental and three technical repeats. ^*^p ≤ 0.05; ^**^p ≤ 0.01; n.s, nonsignificant

As an experimental test, we investigated the mRNA and expression levels of TLR2, TLR4, IL-1β, TNF-α, and NF-κB in human GC using qRT-PCR. Although the highest expression of toll-like receptors is in macrophages and dendritic cells, GC cells transcribe TLR2 and TLR4 more than healthy gastric mucus^[^^27^^,^^28^^]^. Our results showed that the expression of TLR4 was about two times more in the GC samples compared to the normal gastric samples (Fig. 2). However, TLR2 transcripts did not indicate any significant changes in GC samples compared to control group, which emphasizes the possible role of *H. pylori* LPS and TLR4 interaction.

Increased TLR4 expression in GC led to increased LPS-TLR4-MD2 complex formation, which in turn results in elevated PRRs signaling pathway stimulation, ultimately increasing the production of active NF-κB. The upstream sequence of TLR4 has a binding site for NF-κB; thus, NF-κB activation has more positive effect on TLR4 expression^[^^29^^]^. Inflammation-induced LPS signaling alters the invasive and metastatic potential of GC cells^[^^26^^]^. 

Activation of the TLR4 system has been demonstrated to boost resistance to apoptosis in gastric cells. TLR4 can also be up-regulated in many solid tumors, including GC. After binding TLR4 to the LPS of *H. pylori*, by activating MYD88-dependent and non-MYD88 signaling pathways, the transcription factor NF-κB migrates to the nucleus and then raises the transcriptional expression of genes of certain cellular mediators, particularly IL-1β and TNF-α (Fig. 2). 

In computational part of this study, transcriptome analysis conducted on 100 samples confirmed the results of the expression of the key gene for TLR4 pathway (Fig. 1). The expression of those molecules involved in elevation of inflammation including IL-1β, TNF-α, and NF-κB, upregulated, while some anti-inflammatory cytokines, e.g. IL-4, -10, -11, and -13, displayed a pronounce downregulation. 

The overexpression of CXCR7 in GC cells is well documented, and it is associated with tumor development. TNF-α, IL-1β, and bacterial LPS also upregulate CXCL12, the main ligand of CXCR7^[^^30^^]^. Some studies have reported that the blocking of TLR4 reduces CXCR7 expression on GC. TLR4 activation leads to inflammation conditions characterized by a high level of pro-inflammation cytokines production, overexpression of PRRs and co-stimulatory molecules such as MD2, and activation of CXCR7 signaling pathway^[^^31^^,^^32^^]^. In other words, by increasing the resistance of gastric cells to apoptosis, TLRs may cause tumor onset and malignancy. The inflammatory microenvironment generated via MyD88-dependent and independent pathways participates in the overproduction of pro-inflammatory cytokines, i.e. IL-1β and TNF-α and tumor development^[^^33^^,^^34^^]^. Besides increasing the expression of some inflammation-related genes, the polymorphism of many genes was also found to be directly connected with an increased risk of GC. In consistence with our finding, in the GC, TLR4 and downstream signaling molecules have also upregulated. NF-κB, as the main transcription factor in innate immunity, was activated in TLR4 signaling. The overall results of the present study may indicate the key role of the innate immune response in the development of GC. The details of the occurrence and development of GC is not well documented. In this case, the interaction between *H. pylori* and PRRs as a biomarker of inflammation was evaluated.

Altogether, our findings revealed that inflammation conditions occur in GC through upregulation and downregulation of pro- and anti-inflammatory cytokines.

## CONFLICT OF INTEREST

None declared.
